# Diseased Meat

**Published:** 1870-12

**Authors:** 


### DISEASED MEAT.

When poultry or other meat is fresh and good, it has a firm, hard feel, and is elastic ; tainted meat feels soft, and returns to its shape slowly when indented, like dough when pressed with the finger. Fresh meat, well killed, does not moisten the finger ; tainted meat does, and has a slimy feel. Keeping meats until they are about "to turn," makes them tender to eat, but they are harder to digest than fresh meat.

